# Distress, resilience, and self-repair: a topic modeling analysis of Chinese doctoral students' self-disclosure on Xiaohongshu

**DOI:** 10.3389/fpsyg.2026.1921590

**Published:** 2026-10-16

**Authors:** Qian Yin

**Affiliations:** Institute of Language Sciences, Shanghai International Studies University, Shanghai, China

**Keywords:** Chinese doctoral students, corpus-assisted discourse studies, higher education in China, LDA topic modeling, self-disclosure, social media

## Abstract

The rapid expansion and massification of higher education in China have profoundly reshaped doctoral education, subjecting doctoral students to a series of structural challenges. While existing research predominantly employs external analytical perspectives through interviews or questionnaires, limited attention has been paid to Chinese doctoral students' spontaneous self-disclosure and identity negotiation on social media. To address this gap, drawing on 9,903 posts retrieved from Xiaohongshu, this study adopts a Corpus-Assisted Discourse Studies (CADS) approach combined with Latent Dirichlet Allocation (LDA) topic modeling to examine the thematic patterns, doctoral identity construction, and structural dilemmas embedded in online doctoral discourse. Four major topics were identified: supervisor dominance and survival narratives, psychological distress and existential dilemmas, competence formation and academic production, and career-planning and job-seeking preparation. The findings reveal that Chinese doctoral students' self-disclosure on Xiaohongshu is prominently shaped by asymmetrical supervisory relationships, concerns regarding mental wellbeing, publication-centered academic evaluation systems, and increasingly uncertain labor market conditions. Within these constraints, doctoral students discursively construct themselves as emotionally vulnerable yet adaptive subjects who continuously negotiate their identity, self-worth, and future trajectories. Meanwhile, the findings suggest that social media provides a digital discursive space for doctoral students to articulate attempts at emotional self-repair, collective identity formation, and resistance against structural pressures. By foregrounding Chinese doctoral students' spontaneous online self-disclosure on social media, this study offers a bottom-up perspective on their lived experiences and the complexities of doctoral identity negotiation within contemporary Chinese higher education.

## 1 Introduction

Against the background of the national innovation-driven development strategy and the transition toward a knowledge-based economy, higher education in China is undergoing an unprecedented phase of massification in recent decades (Horta and Li, 2023; Shen et al., 2018; Mok and Marginson, 2021). According to the latest statistics, the enrollment of doctoral students in China exceeded 170,000 in 2024, with a total of nearly 680,000 doctoral students in school (Ministry of Education of the People's Republic of China, 2025). On the one hand, this rapid expansion responds to the demand for high-level talent in knowledge production, scientific progress, economic growth and societal development (Levecque et al., 2017; Li et al., 2022; Stamou, 2017). On the other hand, it signifies a notable rise in the doctoral community's social visibility as a knowledge labor group, accompanied by high social expectations that obscure the structural challenges faced by this group.

The expansion of doctoral education in recent decades has significantly changed the global academic labor market, resulting in multiple pressures on doctoral students that have attracted scholarly attention, including highly intensive academic tasks (Bai et al., 2025), stringent publication requirements (Horta and Li, 2023), high rates of delayed graduation and attrition (Tuma et al., 2021; Feizi et al., 2024), degree devaluation and employment difficulties (Servage, 2009; Shen et al., 2018; Jing et al., 2026), and a high incidence of mental health issues (Levecque et al., 2017; Evans et al., 2018). While existing studies provide important references for understanding this group, many of them take an external analytical perspective through questionnaires or interviews. Comparatively, doctoral students as discursive agents and their spontaneous self-disclosure remain largely overlooked.

With the rise of digital technologies, social media platforms have become important spaces for people to express feelings and construct identities (Ye and Gao, 2025; Chu and Jiang, 2024). Their anonymity encourages greater self-disclosure (Schlosser, 2020), enabling doctoral students to freely express their emotions and real situations beyond the limitations of face-to-face communication. Among these platforms, Xiaohongshu is a lifestyle-oriented Chinese social media platform that is roughly comparable to Instagram in its emphasis on user-generated content. Its unique community culture that emphasizes personal experience and everyday narrative sharing has fostered a proliferation of self-disclosure posts, serving as discursive sites where doctoral experiences are interpreted, self-perceptions are negotiated, and complex identities are constructed.

Current studies have explored various aspects of doctoral life but lack a systematic investigation into natural doctoral self-disclosure on social media particularly in Chinese settings. To address these gaps, this study adopts Latent Dirichlet Allocation (LDA), an unsupervised probabilistic topic modeling developed by Blei et al. (2003), to identify latent thematic patterns in Chinese doctoral students' Xiaohongshu posts, followed by a qualitative analysis of representative examples. By foregrounding the doctoral community's own voices and providing a new perspective on their lived realities, this study challenges and deconstructs the oversimplified or stereotypical representations of this group. Moreover, it may help raise public awareness of doctoral students' wellbeing and offer empirical insights for institutions and policymakers to refine doctoral training systems and support mechanisms. Specifically, this study aims to answer the following research questions:

RQ1: What topics characterize Chinese doctoral self-disclosure on Xiaohongshu?

RQ2: How do these topics reflect Chinese doctoral identity construction?

RQ3: What structural tensions in Chinese doctoral education do these topics reveal?

## 2 Literature review

### 2.1 Studies on Chinese doctoral students

Since the promulgation of the Regulation of Academic Degrees of the People's Republic of China in 1980, doctoral education in China has experienced rapid development (Shen et al., 2018). A transformative turning point occurred in 1998 with the initiation of higher education expansion and world-class universities building driven by the people's need for access to higher education and the country's need for economic development, which marks the transition from an elite model to a massified system (Mok and Marginson, 2021). However, this expansion has also generated profound structural consequences that mirror global shifts in the academic labor market (Passaretta et al., 2019), producing an oversupply of PhD graduates relative to available academic positions and heightened competition (Gu et al., 2018; Jing et al., 2026). These pressures are further compounded by systemic factors, such as a rigid academic stratification system (Liu et al., 2024), an intensifying “publish or perish” culture (Wu, 2025), ageism and gender bias in the labor market (Horta and Li, 2024; Bai et al., 2025), and increasingly strict recruitment standards (Horta, 2018), all of which lead to a precarious survival environment and psychological strain faced by PhD candidates. Against this background, a growing body of research has delved into various aspects of Chinese doctoral students' experiences that will be reviewed in the following.

Publication pressure and its impact have become the focus of many studies. Existing research points out that the escalating pressure to publish papers, especially the mandatory requirement to publish in international journals (Xu, 2020), is common in China's academic community, particularly the doctoral student group, and is driven by institutional incentives and assessment systems (Lu, 2022). This publishing-dominant culture not only leads to a power imbalance between supervisors and novices, as well as the disempowerment of novices (Li, 2016), but also affects doctoral students' identity trajectories (Horta and Li, 2023), fosters academic misconduct (Wu, 2025), and risks the homogenization of knowledge production (Xu, 2020). However, current studies tend to approach publication pressure from a macro-structural or outcome-oriented perspective, with limited attention to doctoral students' cognitive and affective experiences within the publication process itself.

The landscape of doctoral career development in China has shifted significantly, with research increasingly highlighting a trend toward diversified career expectations and intentions (Gu et al., 2018; Bao et al., 2020), reasons why doctoral graduates take up non-academic jobs (Chen, 2021), the identity development of PhD graduates transitioning into non-researcher roles (Li and Horta, 2024), and considerations of employment sectors and cities (Chen et al., 2024). Scholars further examine how career trajectories of doctoral students in China are influenced by social and personal factors, including class background and social origin (Li, 2025), ageism in the academic labor market (Horta and Li, 2024), and gender differences in job preference and career development (Shen et al., 2018; Tang et al., 2025). Nevertheless, insufficient attention has been paid to how doctoral students perceive and narrate the transition from academic training to the labor market under conditions of uncertainty and competition, as well as how they negotiate the structural constraints in their career decision-making processes.

The construction of academic identity among doctoral students is understood as a multidimensional process situated at the intersection of personal, institutional, disciplinary, and sociocultural contexts, in which individuals are recognized by self and others of possessing a sense of competence, autonomy, and agency within academic communities (Choi et al., 2021). Studies on Chinese doctoral students elaborate on the factors influencing academic identity formation from different dimensions. For instance, Zhang and Wang (2025) identify contextual, interactive, and psychological dimensions as key forces shaping academic identity, demonstrating how doctoral students negotiate their positioning within China's sociocultural and institutional environment. Zhang et al. (2024) explore the psychological activities involved in doctoral students' understanding of their academic identity that is shaped by the sense of academic meaning and efficacy. Adopting an emic and narrative lens, Sun (2022) reveals how doctoral students (re)position themselves into multi-voiced identity. Ye and Edwards (2017) focus on the identity formation of Chinese PhD students studying abroad. The role of supervision has also been investigated (e.g., Lai and Dai, 2025; Bao et al., 2025). While scholarly focus is mostly on academic or professional identity, the multiplicity and heterogeneity of doctoral identities shaped by intersecting social settings and roles, as well as the internal diversity within this group, are underexplored.

Research on doctoral supervision in China has primarily examined supervisory practices as embedded within the training process, with particular attention to publication pressure, identity development, and mental health. For example, Li (2016) suggests that strong student dependence on supervisors in meeting stringent publication requirements can result in the disempowerment of novice researchers. Extending the focus to identity formation, Lai and Dai (2025) demonstrate how different supervisory styles shape doctoral students' academic and professional identities as well as their epistemic agency, with the high-support, high-structure model proving encouraging. Similarly, Bao et al. (2025) uncover the interactional mechanisms by which supervisors mediate doctoral academic identity construction, including transformative criticism, strategic recognition, and supervisor's own identity as a role model. Liu et al. (2019) find that supervisory relationships mediate the correlation between research self-efficacy and depression/anxiety. Despite these insights, existing studies regarding doctoral supervision in Chinese settings mainly treat it as a contextual or influencing factor within doctoral training, rather than as a primary object of inquiry on its own right. Consequently, insufficient attention has been paid to supervisor-student relationship as an inherently asymmetrical power structure (e.g., Friedensen et al., 2024), and how such relational dynamics shape doctoral students' wellbeing (e.g., Becerra et al., 2021), research productivity (e.g., Khuram et al., 2023), and career trajectories (e.g., Curtin et al., 2016), as well as to negative supervisory experiences (e.g., Tuma et al., 2021).

Scholars have also investigated the mental health of doctoral students in China and its associated factors. For instance, using questionnaires, Liu et al. (2019) report that a considerable proportion of doctoral students show signs of depression and anxiety, with grade in school, frequency of supervisory interaction, publication difficulty, and challenges in balancing work, family, and study identified as associated factors. Through questionnaires and interviews, Bai et al. (2025) explore the mechanisms of anxiety and intervention among different categories of doctoral students, and identify the key influencing factors including gender, age, marital pressures, and self-perception. From the perspective of social support, Cao et al. (2025) develop and validate a four-dimension scale encompassing institutional, supervisory, peer, and family support. While existing studies have made contributions in understanding the psychological state of Chinese doctoral students, they predominantly rely on data elicited through researcher-prompted instruments such as questionnaires, scales, and interviews, with doctoral students' spontaneously produced, naturally occurring self-disclosure remaining overlooked.

### 2.2 Social media and identity construction

In studies on social media, self-disclosure and self-presentation represent two distinct yet interrelated mechanisms of information management. Self-presentation refers to a goal-directed activity of strategically controlling information to influence the audience's impressions about the self that could be true or false (Johnson, 1981; Schlenker and Wowra, 2003). In contrast, self-disclosure is conceptualized as the voluntary communication of inner thoughts, emotional experiences, and personal information with others (Derlega et al., 1993), and it involves the transmission of personal facts, regardless of the self-image created (Johnson, 1981). A specific subtype is distress disclosure that occurs when individuals disclose personal stress, unhappiness, or distress (Coates and Winston, 1987), and the phenomenon of publicly expressing such states online is referred to as distress disclosure on social media (Ye and Gao, 2025).

Unlike strategic self-presentation aimed at impression management, the doctoral discourse examined in this study leans toward authentic self-disclosure, particularly through the sharing of life events or psychological distress, which often involves emotional vulnerability, identity exploration, or moments of personal significance (Saha et al., 2021). While such behavior is considered a mechanism for coping, connecting, and seeking support that may possess therapeutic potential and improve mental wellbeing (Chu and Jiang, 2024; Saha et al., 2025; Ye and Gao, 2025), some scholars find that it may aggravate negative emotions and lead to depressive symptoms (Feinstein et al., 2013; Morina et al., 2024).

Many studies have examined self-disclosure on social media, focusing on its motivations, influencing factors, or psychological consequences. For example, Zani et al. (2022) provide a systematic mapping of motivations underlying self-disclosure on social media, while Şurariu et al. (2025) show that both self-disclosure and self-presentation serve to fulfill users' needs for status and affiliation. Schlosser (2020) suggests that digital characteristics such as anonymity and reduced information richness tend to increase self-disclosure, whereas Cao et al. (2024) demonstrate that social media trust positively affects self-disclosure and ostracism exerts a negative influence. Furthermore, the act of sharing life events on social media is found to exert a protective effect on mental wellbeing by increasing positive affect and sleep quality while reducing negative affect, stress, and anxiety (Saha et al., 2025). In addition, Chu and Jiang (2024) develop a moderated mediation model to demonstrate the mediating role of social media distress disclosure on the relationship between distressors and perceived stress. Despite these advances, current research predominantly samples broad user populations without sufficient differentiation across specific social groups, such as the high-pressure doctoral students, whose disclosure practices may be shaped by unique institutional constraints and identity negotiations. Moreover, limited attention is paid to the thematic substance and meaning-making process embedded in self-disclosure from a discursive perspective.

Furthermore, current research has examined doctoral students' self-presentation and self-representation on social media, highlighting social media platforms as important sites for them to shape professional identity, resist gender stereotypes, and challenge social biases. For example, female PhDs in China have been found to employ diverse strategies on social media platforms such as Zhihu and Xiaohongshu to challenge gender stereotypes and resist biased media portrays (Liu, 2023; Zhang et al., 2024). Beyond gender issues, Wang and Bhatt (2025) reveal the semiotic ideologies in self-naming practices among Chinese doctoral students studying abroad to perform or conceal their doctorateness within cross-cultural environments. In terms of professional identity construction, Wu and Carroll J (2024) show the importance of social networking sites for PhD students in information exchange, gaining support, and developing professional identities, while Radford et al. (2020) investigate both the opportunities and risks associated with constructing scholarly identity on online platforms. Despite these valuable insights, several limitations remain. First, while gendered self-representation of doctoral students is addressed, insufficient attention is paid to the general doctoral population and their shared structural challenges. Second, prior studies tend to prioritize academic or professional identity, leaving the multiplicity and fluidity of doctoral students' identities across personal, emotional, institutional, and social domains relatively overlooked. Third, the reliance on small-scale samples especially in specific disciplines could limit the generalizability of findings.

## 3 Methodology

### 3.1 Data collection

The data for this study were retrieved from Xiaohongshu (also known as the Little Red Book, RedNote, or RED), a popular Chinese social media platform that integrates content sharing (texts, images, and short videos), social networking, and e-commerce. Recent platform analytics indicate that its user base is predominantly young and heavily concentrated in urban centers, with over 83% of active users aged 18–34 (Qiangua Data, 2026). As Xiaohongshu does not mandate public disclosure of personal information, communities are not presented explicitly, but rather hashtag-mediated. Users locate content of interest by keyword search, which returns posts that carry corresponding hashtags. On this basis, the platform's algorithm continuously learns from users' browsing, liking, and following behaviors, and subsequently delivers personalized, hashtag-matched recommendations. Characterized by the user-generated, experience-oriented content and relatively inclusive community culture, Xiaohongshu provides a semi-anonymous digital space that encourages users to engage in spontaneous self-disclosure and seek emotional resonance.

Under this platform-specific background, this study adopted a hashtag-based corpus collection strategy, since hashtags not only signal the poster's self-positioning, community affiliation, and the thematic orientation of the post, but also attract other users who share similar concerns or belong to the same group. This feature makes hashtags especially useful in identifying posts produced by and oriented toward a specific population. Accordingly, the hashtags #读博 (do/pursue a PhD) and #博士生 (PhD/doctoral student) were used as search terms due to their strong associations with doctoral experiences and identities, while video posts were excluded. This sampling strategy prioritized delimiting the target population rather than restricting thematic scope, thereby allowing for a broad capture of diverse doctoral experiences. It should also be noted that Xiaohongshu's built-in result filtering interface does not support user-defined time-range filtering. Search results were then sorted and collected by prioritizing posts according to numbers of likes, comments, and collections, as such engagement metrics reflect shared concerns, emotional resonance, and thematic representativeness.

Data collection was conducted in February 2026, capturing 10,174 posts. Following this initial collection, posts were manually screened. Specifically, commercial advertisements and irrelevant posts by prospective applicants, graduates, supervisors, consultants, institutional accounts, content creators, or other non-target users were excluded. A post was retained only when the post itself or the poster's publicly available profile indicated that the poster was currently enrolled as a doctoral student, and the post contained the poster's own doctoral experiences, personal reflections, emotional disclosures, or experience-based advice directly related to doctoral life. However, it should be acknowledged that verifying user status solely through publicly available self-reported information carries inherent limitations due to the semi-anonymous affordance of Xiaohongshu. A final corpus of 9,903 valid posts was built. The retained posts were published between 2020 and 2026, with more than 95% concentrated in the last 3 years (2024–26), reflecting the recent and contemporary discursive dynamics of Chinese doctoral life. On average, each post received over 410 likes, 168 collections, and 87 comments, indicating substantial peer interaction. For each retained post, the title and text were merged to construct a corpus of Chinese doctoral discourse on Xiaohongshu. All identifiable personal information was anonymized to protect user privacy.

### 3.2 Data analysis

This study adopts a sequential, LDA-led and corpus-assisted analytical design that combined probabilistic topic identification with qualitative contextual interpretation. Corpus-assisted discourse studies (CADS) is an approach that represents a strategic synthesis of corpus linguistics (CL) and critical discourse analysis (CDA) (Partington, 2004; Baker, 2006). While CDA aims to study connections between language, power, and ideology, and reveal how social power, dominance, and inequality are enacted, reproduced, and resisted by text and talk (Fairclough, 1995; Van Dijk, 2001), CL provides an important complement by employing computational tools to identify recurrent linguistic patterns in large corpora (Cheng, 2013), such as frequencies, collocations, and concordances. CADS therefore emerged as a methodological framework that brings together the critical qualitative interpretations of CDA and the quantitative, data-driven strengths of CL, thus facilitating both depth and breadth of analysis (Morley and Bayley, 2009). In addition to conventional corpus techniques, Latent Dirichlet Allocation (LDA), as an unsupervised probabilistic model designed to extract latent topics by detecting patterns of word distribution across large amounts of text (Blei et al., 2003), is increasingly used in discourse studies (e.g., Chen, 2025; Liang and Wang, 2025). In this study, LDA was applied to identify topics and assign keywords, whereas CADS contextualized the model-generated topics and keywords by examining collocational networks and concordance lines.

The analytical procedures include python-based text preprocessing, topic modeling, and discourse analysis assisted by high-frequency collocations and example sentences. First, the raw Chinese textual data were preprocessed using Python. Unlike English, Chinese text is written without spaces between words, and therefore jieba (0.42.1), a Python library for Chinese word segmentation, was adopted to convert the raw text into token sequences for subsequent processing. Regular expressions were used to remove noise, such as emojis and URLs, and tokens included in the Sichuan University Machine Intelligence Laboratory stop-word list were subsequently removed. Then, LDA was applied to uncover the latent semantic structures^1^. The four-topic solution was selected using the elbow method as shown in Figure 1^2^. For each topic, the top 30 keywords with the highest topic-specific probabilities were extracted (see Table 1), and an Inter-topic Distance Map visualizing topic prevalence and similarity via multidimensional scaling was generated (see Figure 2). In this visualization, each bubble represents a topic, with the size of the bubble reflecting topic prominence and the distance between bubbles indicating inter-topic similarity. Subsequently, the keywords identified through LDA were used as search terms in Antconc to retrieve high-frequency collocations and concordance lines as examples, providing recurrent lexical patterns and their contexts to assist the qualitative interpretation of the doctoral discourse. Notably, the qualitative examples for each sub-topic were sampled from posts with the highest density and co-occurrence of topic-specific keywords, thereby capturing keyword-rich illustrations most semantically representative of their respective topics.

**Figure 1:**
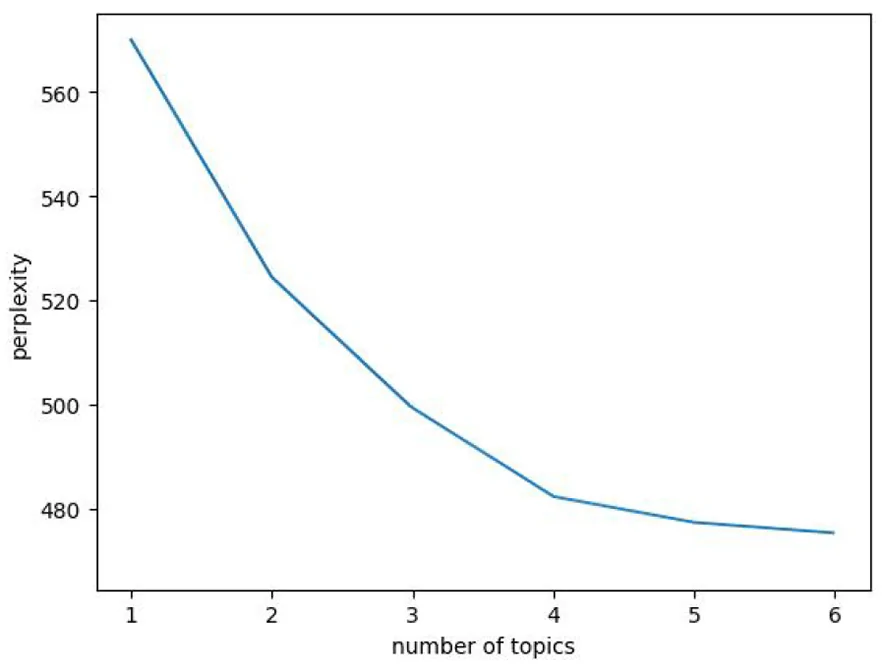
Perplexity curve.

**Table 1:** Topics and top-30 keywords.

Topic 1	Mental state, supervisor, scientific research, mindset, paper, graduation, graduate student circle, group meeting, worry, life, fear, delay, dominated, get along, teacher, experiment, drop out, academic, work, pursuing PhD, female PhD, dissertation, laboratory, report, adjust, senior male student, senior female student, boss, despair, progress
Topic 2	Anxiety, lifestyle, pursuing PhD, pressure, life, feel, time, emotion, mindset, effort, state, self, study, paper, meaning, suffering, experiment, friends, hope, like, problems, growth, see, breakdown, face, persist, doubt, internal friction, body, failure
Topic 3	Paper, supervisor, research, scientific research, academic, literature, ability, learning, time, writing, method, field, data, core, publish, experiment, project, achievement, logic, theory, article, thinking, goal, read, journal, submission, knowledge, discuss, cognition, revision
Topic 4	Job, application, graduation, teacher, school, institution, supervisor, choice, employment, direction, interview, major, requirements, preparation, job seeking, future, peers, university, time, comparison, experience, advice, degree, scientific research, domestic, college, position, resume, materials, recruitment

**Figure 2:**
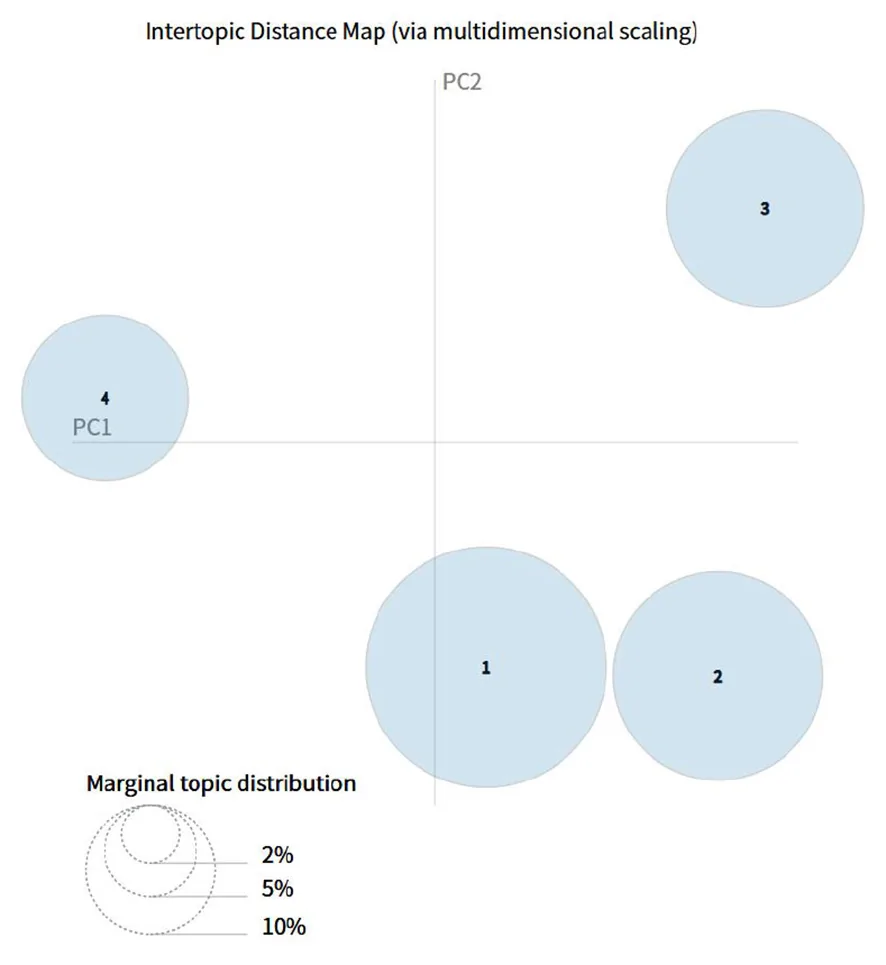
Intertopic distance map.

## 4 Results

As shown in Table 1, four salient thematic clusters were identified, which cover distinct yet interrelated dimensions of doctoral experiences in the context of contemporary Chinese higher education, including (1) supervisor dominance and survival narratives, (2) psychological distress and existential dilemmas, (3) competence formation and academic production, and (4) career planning and job-seeking preparation. These four clusters address RQ1 by identifying the major topics that characterize Chinese doctoral students self-disclosure on Xiaohongshu.

### 4.1 Topic 1: supervisor dominance and survival narratives

This topic mainly presents doctoral students' positioning within supervisor-dominated academic structures, centering on supervision, research governance, graduation requirements, and mental state. Among the keywords, one of the most salient terms “supervisor” and its high-frequency collocations “dominated” and “fear” together form the high-frequency expression “fear of being dominated by the supervisor”. It depicts a daily routine that is “dominated” and filled with “fear” and “worry,” suggesting a discourse field organized by institutional authority and hierarchical relations, in which the agency of doctoral students is constrained. Specifically, keywords under Topic 1 can be further divided into 4 sub-topics, institutional network, regulatory mechanism, negative outcomes, and emotional response.

#### 4.1.1 Institutional network

Keywords including “supervisor,” “teacher,” “boss,” “senior male/female student,” “laboratory,” “graduate student community” constitute sub-topic 1, institutional network, which reflects a relatively closed academic field with a clear hierarchy. The keyword “boss” within Topic 1, as illustrated by Example 1, invokes a transactional metaphor for supervisory relationships and a perceived shift of the supervisor's role from a mentor that provides guidance, career support and psychosocial support (National Academies of Sciences, Engineering, and Medicine (NASEM), 2019) to an employer providing capital in exchange for labor, as well as the students' identity from a mentee to an academic laborer. Many doctoral students in the collected posts perceive the inherent power imbalance in the supervisor-student relationship, feeling exploited and reduced to cheap labor.

(1) *6,946: When working on research projects, I just regard myself as a company employee and adopt the mindset of a wage-earner. I finish what my boss asks me for as quickly as I can, so that I can have extra time to work on my own stuff*.

#### 4.1.2 Regulatory mechanism

Keywords “group meeting,” “report,” “progress,” “paper,” “dissertation,” “experiment,” and “work” collectively construct the group meeting as an institutionalized mechanism of monitoring research productivity and evaluating academic performances in doctoral training. Specifically, “group meeting” frequently co-occurs with expressions such as “weekly,” “each time,” “times,” “frequency,” “report,” and “progress”; “report” frequently collocates with “progress,” “regularly,” “preparation,” and “nervous”; and “progress” is often paired with “slow,” “lagging,” “stagnation,” “urge,” “push,” “follow up,” “anxiety,” and “others.” As Examples 2 and 3 suggest, group meetings are perceived by many students as high-frequency, high-pressure events accompanied by extensive preparation and persistent anxiety, where they must quantify and publicly present their research progress in a collective evaluative environment, and have to be assessed and compared with other group mates. When progress is slow or stagnant, students often feel heightened anxiety and self-doubt.

(2) *164: I don't want any interactions with my supervisor. Just seeing his WeChat messages makes me extremely anxious. The weekly group meetings are way too much, and even if I'm not the one being called out, I feel incredibly nervous and stressed every time I see him*.
(3) *5,450: I feel like there is a huge gap between me and other students after every group meeting. I'm always caught in a vicious loop of anxiety, depressions, and self-doubt, leading to endless internal friction. Pursuing this PhD is truly miserable!*

#### 4.1.3 Negative outcomes

Terms including “dominated,” “get along,” “delay,” “drop out,” “graduation,” and “female PhD” form a subtopic concerning perceived or anticipated negative outcomes during doctoral study. Within the corpus, these terms occur in contexts that discursively associate supervisory control, academic pressure, and institutional uncertainty with concerns about delayed graduation, dropout, diminished self-worth, and gendered employment anxiety. Among these keywords, “dominated” often collocates with “supervisor,” “being,” and “fear,” and “get along” frequently co-occurs with “supervisor,” “how,” “mode,” and “way.” These lexical patterns point to a perceived loss of autonomy and the asymmetry of power in which doctoral students conceive the supervisor-student relationship as an uncertain negotiation that requires constant communicative strategy adjustments.

The keyword “graduation,” frequently associated with “on time,” “meet,” “requirements,” “conditions,” and “delayed,” is constructed as a potentially unattainable goal that is conditional upon evaluative metrics rather than an anticipated terminal point. While “delayed graduation” that collocates with “years,” “risk,” “facing,” “reasons,” and “unwilling” is connected with psychological erosion and identity crisis in Example 4, dropping out is framed as a response to sustained self-deprecation and lack of positive feedback in Example 5. In addition, the keyword “female PhD” and its collocates such as “job-seeking,” “stereotypes,” and “age,” introduce a gender dimension to this subtopic. Example 6 suggests that female doctoral students are positioned at the intersection of academic pursuit, career expectations, and social norms, and their identity becomes a liability in the labor market.

(4) *5,049: The hardest part of a delayed graduation is the collapse of one's self-valuation. We all started our PhD journey with the confidence and pride accumulated from years of academic excellence. However, negative feedback can snowball over time, growing larger and larger until, at some point, it becomes the final straw that breaks the camel's back*.
(5) *5,444: The thought of dropping out keeps circling in my mind and is driving me crazy. Perhaps I'm just not cut out for research? There is something wrong with my mindset. I can't handle prolonged failure without any positive feedback*.
(6) *4,781: As a 35-year-old unmarried female medical PhD candidate, I was rejected by another hospital during interviews. The reason? My marital status. They claimed dating would drain half my energy, while getting married and having children would consume the rest, leaving no time for research. Fine. I'm guilty. It's all my fault*.

#### 4.1.4 Emotional response

This dimension that includes “fear,” “despair,” “worry,” “mental state,” “mindset,” “adjust,” and “life” describes doctoral students' psychological strain and self-regulation under hierarchical governance and institutional pressure, implying a form of emotional release and psychological repair through discursive practices on social media. On the one hand, “fear,” “despair,” and “worry” are often triggered by supervisory control and graduation uncertainty. Specifically, “fear” frequently co-occurs with expressions such as “the fear of being dominated by the supervisor,” “future” and “uncertainty,” and “worry” frequently co-occurs with “graduation,” “employment,” and “delay,” indicating that doctoral students' anxiety emerges at the intersection of academic completion and labor market uncertainty (Example 7) and stems from binding their self-worth entirely to supervisors' evaluations and external standards (Example 8). On the other hand, “mindset” with its collocates such as “adjust,” “maintain,” “calm,” “positive,” “optimistic” and “open,” and “life” with its collocates such as “balance,” “research,” “pace,” “enjoy,” “simple,” and “happy,” reflect doctoral students' attempts to mitigate psychological strain in discourse on mindset adjustment and work-life balance (Example 9).

(7) *1,937: At the peak of my anxiety, I couldn't sleep at all. I worried about graduation, about future employment, about my efforts being in vain, and about my research output not being outstanding enough*.
(8) *2,579: When our self-worth becomes tightly bound to data, results, and supervisors' evaluations, the boundary between the self and external standards becomes blurred, and we surrender our self-judgement to outside forces. Under such conditions, one's emotions become highly unstable, swinging from joy at a positive comment to frustration at a negative one. This also reflects an acute sensitivity to the power dynamics inherent in the supervisor-student relationship*.
(9) *2,011: Throughout the long, arduous process of doing research, especially when there is little external recognition, it is crucial to build an internal sense of achievement and confidence. We must adjust our mindset and approach the PhD journey as a marathon. It is essential to take scheduled breaks and maintain a balance between research and life*.

Collectively, Topic 1 constructs doctoral experience as shaped by asymmetrical power relations, where research progress, performance evaluation, and even degree acquisition are contingent on the supervisory framework. This topic reveals a discursive field where a stratified institutional network defines power relations, regulatory mechanisms impose continuous monitoring, and these structural pressures collectively produce negative outcomes and induce intense emotional responses. During the process of distress disclosure, doctoral students strive to adjust their mindset and reclaim their self-worth.

### 4.2 Topic 2: psychological distress and existential dilemmas

The second topic reflects doctoral students' emotional experience and the deep, underlying psychological struggles. Frequent references to “anxiety,” “pressure,” “suffering” and “breakdown” indicate the intensity of their affective strain, and expressions like “life,” “meaning,” “self,” “doubt” and “failure” signal their constant self-doubt and repetitive scrutiny of the meaning of pursuing a PhD. However, in this topic, these negative experiences are often contrasted with keywords such as “growth,” “persistence,” “hope,” “mindset,” and “effort,” suggesting a discursive mechanism through which pressures are reinterpreted as opportunities for personal growth and character building. There are three sub-topics under topic 2: mental and physical symptoms, existential crisis, and coping strategies.

#### 4.2.1 Mental and physical symptoms

This dimension that includes “anxiety,” “pressure,” “emotion,” “suffering,” “breakdown,” “internal friction,” and “body” captures the escalation from external pressure to internalized anxiety, emotional dysregulation, and eventual psychosomatic symptoms reported in the doctoral discourse on Xiaohogshu, as demonstrated by the collocates of “anxiety,” such as “sleep deprivation,” “insomnia,” and “depression,” and the symptoms like “hair loss,” “accelerated heart rate,” and “palpitations” in Example 10. In addition, the keyword “body” is frequently collocated with terms of exhaustion, such as “overdrawn,” “collapsed,” and “depleted,” and Example 11 describes doing a PhD as “physical trauma” and “spiritual death,” suggesting that the acquisition of the doctoral degree is at the expense of both mental and physical wellbeing.

(10) *3,823: Since starting my Master's, I've been trapped in persistent internal friction and depression. During my PhD application and my doctoral studies, I have experienced frequent anxiety, insomnia, and hair loss. A medical check-up revealed several abnormal indicators, along with somatic symptoms such as an accelerated heart rate and palpitations*.
(11) *4,274: Doing a PhD has truly transformed me from a cheerful, optimistic girl into someone who's insecure, sensitive, and consumed by internal friction. It really is a comprehensive ordeal: a minor upgrade in credentials, but at the cost of the disfigurement of personality, severe physical trauma, spiritual death, and the ruin of a career*.

#### 4.2.2 Existential crisis

“Life,” “meaning,” “self,” “doubt,” and “failure” constitute this sub-topic that signals a shift from immediate emotional distress to deeper, reflexive contemplation of broader existential questions about personal identity, the purpose of research, and the meaning of life. This discourse of existential inquiry around the keyword “meaning” features an interrogative tone or negated forms, frequently collocated with “what is the meaning of doing a PhD?,” “where is the meaning,” “what on earth,” “no meaning,” “meaningless,” and “at all.” As Example 12 illustrates, doctoral students often experience a sense of void characterized by feelings of emptiness, confusion, and a lack of direction when they realize that their research fails to contribute to society or personal growth. Moreover, “doubt,” “failure,” and the fact that “self” is overwhelmingly paired with negative verbs like “doubt,” “negate,” “sink into,” and “attack,” reflect a loss of self-efficacy under rigorous academic evaluation systems. In Example 13, the dominance of a monolithic, publishing-based evaluative system leads to the suppression of other value frameworks, resulting in a narrowed sense of self-worth and eventual emotional numbness. Example 14 frames failure as a norm that leads to the gradual erosion of prior self-perceptions.

(12) *3,351: What is the meaning of doing a PhD? Recently, I've once again fallen into a state of emptiness. Has the world become a better place because of my research? No. Have I become a better person because of my research? Also no*.
(13) *1,205: It is a downward spiral: self-doubt, then depression, then dissociation. It starts with deep self-doubt, which makes you feel completely worthless and incapable of doing anything right. This happens because every value system other than “publishing” has been ignored or blocked out. Over time, you sink into recurring depressive states. It becomes increasingly difficult to find a sense of value from any activity other than research, and gradually, interest in many aspects of life fades. Eventually, even when you finally achieve something in your research, you don't seem to feel happy anymore*.
(14) *1,441: What does it mean to do a PhD? It means constantly doubting yourself, enduring countless failures and setbacks until your sharp edges are worn down. It means shedding the illusion of superiority and learning to accept your mediocrity*.

#### 4.2.3 Coping strategies

Keywords including “friends,” “hope,” “like,” “growth,” “mindset,” “effort,” “face,” and “persist” indicate active coping strategies in face of adversity, revealing a discursive tendency to reinterpret suffering as part of the developmental trajectory. “Friends,” often co-occurring with “family,” “chat,” “meet,” “together,” “hang out,” and “weekends,” highlights the interpersonal network as a crucial emotional resource. Example 15 shows that communication with friends and family serves as a channel for emotional release and a means of reconnecting with the world. “Like,” which frequently appears in “things/people/directions/books/life you like,” represents doctoral students' self-regulatory practices to buffer against repeated academic setbacks and restore vitality and intrinsic motivation, as illustrated in Example 16. Keywords “growth,” “effort,” “face,” and “persist” as well as Example 17 reflect cognitive restructuring mechanisms of reframing growth from academic achievement to mental maturity and the importance of self-acceptance and self-affirmation in persisting despite adversity. Taken together, doctoral students' coping operates as a multi-layered process that integrates social support, behavioral adjustment, and cognitive reframing, revealing Xiaohongshu as a vital space for collective encouragement and self-healing.

(15) *4,053: Stay in regular contact with friends and family to help shift your attention. You don't have to bear emotions alone; seeking support can effectively strengthen your self-esteem and confidence, allowing you to reconnect with the world*.
(16) *1,620: Cultivate your interests. Allow yourself to rest, to make mistakes, and to be imperfect. Fully accept and listen to yourself. As long as you stay on track for graduation, make time to do what you genuinely enjoy. You must have a life outside of research to maintain a sense of vitality*.
(17) *3,297: Pursuing a PhD is not only about academic advancement, but also about mental growth and inner maturity. You will face various challenges, but each breakthrough will make you more determined and resilient. It's essential to acknowledge our efforts and celebrate every bit of progress, no matter how small. Such self-affirmation empowers us to persist without being defeated by difficulties*.

In conclusion, Topic 2 depicts the interplay between doctoral students' acute emotional distress, deep existential questioning, and adaptive coping efforts. Although prolonged academic pressure is internalized into anxiety, psychosomatic symptoms, and existential crisis, a narrative of resilience is constructed to counterbalance the predominantly negative discourse through the mobilization of social support, engagement in meaningful activities, and cognitive restructuring.

### 4.3 Topic 3: competence formation and academic production

The third topic revolves around doctoral students' engagement in academic activities. Terms such as “literature,” “read,” “knowledge,” “research,” “data,” “experiment,” “method,” and “theory” emphasize the accumulation of knowledge and the technical details of research work. References to “ability,” “thinking” and “logic” highlight skill acquisition and competence development. Keywords including “paper,” “article,” “publish,” “submission,” and “journal” point to the centrality of research output in doctoral life. Therefore, research productivity is constructed here not merely as a requirement but as the core marker of academic identity and professional worth. This topic is further divided into knowledge input, research process and academic output, and academic evaluation system.

#### 4.3.1 Knowledge input

This sub-topic that includes “literature,” “field,” “read,” “knowledge,” “thinking,” “learning,” “cognition,” and “ability” reflect the initial stage of doctoral training as a foundational and accumulative process of intensive knowledge input, ability development, and habit formation for subsequent academic production. “Literature” (frequently collocated with “read,” “review,” “organize,” “take notes,” “intensive reading,” “collect,” etc.,) and “read” (frequently collocated with “literature,” “massively,” “habit,” “daily,” “widely,” “classical,” “foundation,” etc.,) reflect a highly regular engagement with disciplinary knowledge and the gradual internalization of academic norms. As Example 18 shows, the daily habit of reading literature is perceived as a prerequisite for enriching personal knowledge repository and building a solid academic foundation. In addition, “cognition” and “thinking” that often appears in “critical thinking,” “logical thinking,” “independent thinking” and “deep thinking” are framed as an intellectual ability that needs to be developed during doctoral studies (Example 19).

(18) *1,733: It's important to develop a consistent habit of reading literature. When I first started my PhD, I would intensively read an article from a top-tier journal in my field each day to help construct my knowledge system, while also skimming several others to stay informed about current research trends, gaps, and topics of personal interest. Over time, this habit gradually enriches my knowledge base and broadens my analytical perspectives*.
(19) *1,961: What a PhD offers me is not immediate material gain, but rather a long-term accumulation of abilities. My writing skills, logical thinking, critical thinking, research methods, and innovative capability have all seen significant enhancement. Although these abilities may be difficult to quantify in the short term, I'm confident that they will prove valuable in the future*.

#### 4.3.2 Research process and academic output

Within this sub-topic, the keywords “research,” “scientific research,” “academic,” “writing,” “method,” “data,” “experiment,” “project,” “logic,” and “theory” together indicate the mid-to-late stage of doctoral training as a process of knowledge production and academic writing. “Scientific research” and “research” (often collocated with “field,” “plan,” “method,” “question,” “design,” “content,” “theory,” “idea,” “background,” “project,” and “subject”) capture the systematic procedures of scientific research. As stated in Example 20, doctoral research is framed as an integrated process involving research design, experimental setups, data analysis, and the interpretation of results, and this procedural competence conditions later-stage productivity and output quality. The keyword “writing” functions as the primary site of academic output, often collocated with “paper,” “academic,” “logic,” “thinking,” “ability,” and “training.” Example 21 demystifies writing as a structured and trainable practice and highlights the IMRaD structure as a formalized logical framework. More importantly, as illustrated in Example 22, some doctoral students discursively construct their academic development as a transition from “acquiring knowledge” to “creating knowledge,” framing their emerging scholarly identity around the aspiration of becoming “domain experts.”

(20) *3,832: Doctoral students should be capable of designing sound research plans and experimental protocols. The mastery of appropriate techniques and methods is crucial for performing experimental operations and data processing effectively. In addition, doctoral students need strong scientific writing and communication skills to present research questions, methods, results, and conclusions with clarity and precision*.
(21) *7,434: Academic writing is in fact grounded in a clear and reliable structural logic. Most research articles can be deconstructed into four major components: Introduction, Methods, Results, and Discussion. Establishing this structure in advance and then progressively filling in the content is an effective way to completing a paper*.
(22) *2,924: The core task of a PhD is to create knowledge rather than merely acquire knowledge. While undergraduate and master's education is essentially a form of inherited learning with the main goal of building a knowledge system and mastering disciplinary foundations, the doctoral stage requires breaking through existing frameworks to make original contributions within a specialized field. This transition from recipient to producer represents a qualitative leap in academic competence, making individuals domain experts*.

#### 4.3.3 Academic evaluation system

In this dimension, “paper,” “core,” “publish,” “achievement,” “article,” “goal,” “journal,” “submission,” and “revision” collectively construct an academic evaluation system in which publications, particularly in “core” journals, function as a requirement for graduation and the primary measure of doctoral students' scholarly value. “Paper” and “article” (both frequently collocated with “write,” “submit,” “revise,” “rejected,” “accepted,” and “publish”), as well as “achievement,” together signal the measurable academic output and the result-oriented evaluative logic. Example 23 metaphorically compares them to “lifeline” and “hard currency.” Closely tied to them are the keywords “journal,” “submission,” “revision,” and “publish,” which represent the publication process. As Example 24 indicates, the life cycle of a paper, from initial drafting to submission, peer review, revision, and eventual acceptance, constitutes a prolonged and iterative process marked by external uncontrollable factors and huge emotional and time costs.

(23) *6,445: If you aim to pursue an academic career, your core mission is to produce high-quality, high-impact research outputs. This is your absolute lifeline, the hard currency of academia, and the foremost criterion for evaluating your academic value*.
(24) *2,874: Only those who have experienced it truly understand how difficult it is for PhD students to publish papers. The journal selection and submission stage (typically 1 week to 1 month) requires learning journal preferences, aligning the paper's topic and level with suitable journals, preparing submission materials, and checking formatting details, since even minor oversights may lead to missed opportunities. Then comes the most frustrating and time-consuming review and revision stage (typically 3–12 months). After submitting a meticulously revised manuscript, you may wait months for a response, only to receive an outright rejection or extensive revision comments that adds another 1–3 months. Even when the acceptance finally comes, you don't feel much joy but a sense of relief*.

Topic 3 constructs doctoral training as a multi-stage process. It begins with intensive knowledge input characterized by sustained literature reading and the cultivation of cognitive abilities. This habitual foundation is then transformed through the research process into the production of scholarly knowledge, representing a shift in identity from passive learners to domain experts. Ultimately, these activities are embedded within a publication-centered academic evaluation system, where journal articles serve as the primary indicators of academic value.

### 4.4 Topic 4: career planning and job-seeking preparation

The fourth topic focuses on employment and future planning beyond the academic field. Keywords such as “job,” “employment,” “university,” “direction,” “choice” and “future” reflect doctoral students' decision-making under conditions of uncertainty and the trade-offs between staying in academia or leaving the academia. Terms such as “application,” “interview,” “recruitment,” “position” and “resume” indicate an acute awareness of limited academic positions, competitive labor market and the need for strategic preparation. This topic can be further divided into career planning and job-seeking preparation.

#### 4.4.1 Career planning

In this sub-topic, keywords “job,” “employment,” “choice,” “university,” “institution,” “college,” “school,” “degree,” and “domestic” reflect doctoral students' perceptions of the contemporary employment landscape and their strategic or disillusioned approach to future planning. “Job” (often collocated with “find,” “hard to find,” “stable,” and “satisfactory”) and “employment” (often paired with “grim,” “market,” “prospects,” “pressure,” and “competition”) indicate a shared conception of a tightening and increasingly competitive labor market for PhD students. In Example 25, the employment environment is described as grim, characterized by intensified performance requirements, shrinking positions, and declining material incentives. The keywords “university,” “institution,” “school,” and “college” represent the traditional destination for PhDs, which is now considered a high-threshold, high-risk career path that is no longer guaranteed. While Example 26 highlights escalating entry barriers, institutionalized pressures, and the credential inflation, Example 27 observes a transition toward diversified, non-academic career paths.

(25) *1,106: After spending some time job hunting, I feel that both the current and future employment situation is quite grim. First, performance evaluations are becoming increasingly stringent. The idea of a job that is well-paid, low-pressure, and close to home has largely become unrealistic. Second, universities are not short of candidates, and most institutions have very limited vacancies. Third, housing allowances are decreasing or even disappearing. Fourth, overall compensation and the cost-effectiveness of academic positions are declining. Salary cuts and reduced research incentives are pretty common*.
(26) *4,036: In academia, the “up-or-out” system has become the norm. Entry into top-tier universities now requires a combination of overseas experience and publications in top journals. Meanwhile, in corporate R&D, employers seek practitioners who can generate immediate profit, and many PhDs are ironically rejected for being “overqualified” and lacking hands-on skills*.
(27) *4,678: A clear trend has emerged in recent years, that many exceptional PhD graduates, even those with strong publication records, do not pursue academic careers but venture into the industrial sector. This stands in sharp contrast to the traditional notion that the sole purpose of a PhD is to enter academia*.

#### 4.4.2 Job-seeking preparation

The keywords “job seeking,” “recruitment,” “preparation,” “interview,” “resume,” “materials,” “position,” “requirements,” “experience,” and “advice” represent the preparations required in job seeking, which is depicted as an information-intensive, experience-driven, and strategy-oriented process. Keywords including “recruitment” (often collocated with “information,” “university,” “position,” “announcement,” “post,” “websites,” and “platforms”), “position” (often collocated with “recruitment”, “competition,” “requirements,” “matching,” and “fitting,”) and “requirements” (often collocated with “high,” “meet,” “position,” etc.,) highlight the importance of access to information about recruitment channels and position requirements (Example 28). The keyword “preparation” emphasizes early and systematic planning, involving both material preparation and competence preparation (Example 29). Therefore, job-seeking preparation is framed as a continuous process of capital accumulation and professional identity formation, in which strategic presentation of one's academic achievements is necessary for academic potential proving. Meanwhile, the keywords “experience” and “advice” suggest that Xiaohongshu has become an important experience-sharing platform for doctoral students to seek advice, transmit knowledge, and reduce uncertainty in the face of the complex and ever-changing recruitment environment.

(28) *4,961: Securing a faculty position typically involves resume submission and interviews, with occasional written tests depending on the institution's announcement. The most direct approach is to sell yourself through your supervisor's network. If that's not an option, you'll need to rely on independent applications. Many universities accept resumes year-round via their official websites. Another effective strategy is to bring your papers to academic conferences and forums, where you can interact directly with deans*.
(29) *5,740: Early preparation is essential. (1) Prioritize publishing in top or core journals in your field, supplemented by patents, research projects, and teaching experience. (2) Closely monitor the HR offices and department websites of target institutions to understand evaluation criteria, salaries, and benefits. (3) Refine application materials: keep your CV concise and relevant; align your research statement with institutional priorities; include a teaching plan with sample lessons; and prepare publication records and recommendation letters. (4) During the interview, focus on showcasing your research potential and teaching ability*.

Topic 4 captures how doctoral students' career decision-making and job-seeking practices are shaped by structural constraints and intensifying competition. On the one hand, the growing awareness of a stratified employment landscape prompts a shift from traditional academic aspirations toward diversified, non-academic trajectories. On the other hand, job-seeking preparation is perceived as an information-driven and experience-based process, involving early planning, targeted applications, and the accumulation of academic capital.

## 5 Discussion

The four topics identified in this study construct a comprehensive discursive representation of doctoral life. Topic 1 Supervisor Dominance and Survival Narratives (T1) situates doctoral students within institutional and relational power structure. Topic 2 Psychological Distress and Existential Dilemmas (T2) highlights the psychological consequences of this structure and how individuals negotiate and self-regulate these pressures. Topic 3 Competence Formation and Academic Production (T3) emphasizes competence accumulation and research production as the basis of evaluation. Topic 4 Career Planning and Job-seeking Preparation (T4) moves from doctoral training to the labor market. The results thus suggest that within the examined corpus, doctoral identity on social media is discursively represented at the intersection of external constraints, internal experience, professional practice, and future orientation. This section integrates the empirical findings of section 4 with broader literature to interpret how the identified topics reflect the multi-layered dynamics of doctoral identity and respond to the structural tensions inherent in China's doctoral education system, thereby addressing RQ2 and RQ3.

### 5.1 Oppressed academic laborers in asymmetrical supervisory relationships

T1 foregrounds doctoral supervision as a structurally embedded site of power asymmetry and discursively constructs doctoral identity as being oppressed, subordinate, and disempowered. Recurrent lexical choices depict a subject position characterized by constrained agency, academic dependency, and emotional vulnerability. Drawing on accounts of the academic-scientific field and educational institutions that are constituted by power and domination (Bourdieu, 2004; Reed-Danahay, 2009), the analysis of T1 suggests that some doctoral students articulate an acute awareness of “leader-follower relationships” (Vähämäki et al., 2021), discursively depicting situations where they take instructions to meet pre-defined targets at the cost of sacrificing their academic autonomy and self-worth under hierarchical authority.

The analysis of T1 reflects well-documented structural issues in doctoral education, particularly the deep-rooted power imbalance and hierarchical distribution of roles between supervisors and students (Friedensen et al., 2024; Paulino and Bispo, 2026). The perception of graduation as a conditional and potentially unattainable goal in the corpus may stem from supervisors' documented role as gatekeepers to the qualification and the academic discipline that results in their ownership or even suppression of the final result (Lee, 2008). This gatekeeping role is especially salient in the Chinese context, where doctoral progression and graduation are often heavily dependent on supervisory approval and publication outcomes, thereby creating a survival environment marked by high risk and high uncertainty. Moreover, the discourse around group meetings as high-pressure sites of frequent monitoring and performative evaluation aligns with research findings that more controlling supervision styles may undermine students' academic autonomy (Wichmann-Hansen and Schmidt Nielsen, 2024), and extremely frequent meetings are associated with less positive affect (Becerra et al., 2021).

More importantly, the extreme negative consequences of hierarchical control described in T1 echo a substantial body of literature indicating the negative effects of unsupportive or dysfunctional supervisory relationships on students' physical or mental well-being (Rummell, 2015; Li et al., 2022), productivity (Scandura, 1998), delayed degree attainment (Graham, 2013), and dropout risk (Glorieux et al., 2025). The survival narratives identified in T1 thus reflect not only individual perceptions but also broader systemic tensions within doctoral education, where institutional pressures, supervisory authority, and limited student agency intersect to produce a structurally constrained and emotionally burdened doctoral experience.

### 5.2 Disempowered yet adaptive self-disclosers on social media

In relation to T1, T2 extends the representation of doctoral identity beyond structural disempowerment by positioning some doctoral students as reflective and adaptive subjects who articulate intense psychological distress and existential doubt on social media. As the discursive evidence in T2 suggests, some doctoral students actively engage in cognitive reframing by reinterpreting experiences of suffering as opportunities for personal growth. Such accounts constitute narratives of attempted discursive self-repair through which they seek to reconstruct their fragmented identities. In this sense, they are discursively positioned as subjects attempting emotional self-healing.

The pervasive self-reported anxiety and psychosomatic symptoms identified in T2 resonate with extensive literature evidence that doctoral students are among the most vulnerable groups within the academic ecosystem (Chen, 2025), with disproportionately high levels of stress and mental health problems (Evans et al., 2018; Levecque et al., 2017; Rummell, 2015). As previous studies indicate, the mismatch between elite ideals and reality generates persistent anxiety and self-doubt (Bai et al., 2025), manifesting as what Lovitts (2008) describes as the “silent crisis” of doctoral education. When doctoral students internalize the rigorous, publication-centered evaluation system as the sole measure of self-worth, repeated setbacks and long-term lack of positive feedback gradually erode their sense of academic meaning and academic self-efficacy, both of which are believed to be the driving force and crucial psychological condition for them to form academic identity (Zhang et al., 2024).

Within this context, the examined posts represent Xiaohongshu as an anonymous digital space where doctoral students can articulate otherwise suppressed emotions and engage in collective sense-making as a subtle form of discursive resistance to their perceived disempowerment within the academic system. As prior research suggests, people are motivated to express and share their negative emotions with others when encountering stressful events (Stiles, 1987), and social media affordances such as anonymity (Schlosser, 2020), and reduced (privacy) risk and greater perceptions of benefits (Chu and Jiang, 2024), have been argued to increase self-disclosure, enabling people to freely disclose their otherwise private identities or share controversial content (Schlosser, 2020; Chen and Berger, 2013). The identified discursive practices on Xiaohongshu can therefore be interpreted here as attempts to alleviate psychological distress.

Notably, prior research has conceptualized social media distress disclosure as a potential coping mechanism (Saha et al., 2025). Drawing on the fever model of disclosure (Stiles, 1987), the high frequency of emotional expressions in the collected posts can be seen as an acute response to psychological distress that simultaneously contributes to emotional recovery. Moreover, a significant body of research has reported the therapeutic potential of social media self-disclosure in improving wellbeing, enhancing sleep quality, promoting self-acceptance, and reducing stress (Saha et al., 2025; Luo and Hancock, 2020; Towner et al., 2022). Against this theoretical background, collective venting and shared narratives of suffering on Xiaohongshu could theoretically create a “protective effect” (Saha et al., 2025), functioning as discursive resources through which users seek emotional relief, foster mutual recognition, and negotiate a shared group identity centered on vulnerability, resilience, and community belonging.

### 5.3 Frustrated research engines in the publication-centered academic system

The discourses of knowledge acquisition, research output, and academic evaluation in T3 construct a doctoral identity characterized by a transition from being a learner to being a researcher (Zhang et al., 2024), revealing how doctoral training functions as a process of academic socialization and professional identity formation (Sinclair et al., 2014). Existing research has shown that publishing is a critical factor shaping young scholars' professional development and career opportunities (Sverdlik et al., 2018; Dai et al., 2023). However, this academic or professional identity formation process is distorted by the dominance of publication-centered evaluation systems, in which doctoral students' personal value, professional legitimacy, and future prospects are tightly bound to measurable scholarly outputs.

As publications become the hard currency (Kennedy, 1997), doctoral students are compelled to compete for limited resources (Wu, 2025), reducing the doctoral journey to an academic competition and peers to rivals rather than collaborators (Horta and Li, 2023). As a result, under the high-pressure “publish or perish” environment, the aspiration of “science as a vocation” has been replaced by “publishing as a vocation” (Wu, 2025), and doctoral students are increasingly viewed as research engines (Sampson and Comer, 2010). As the growing demand for publishing space from doctoral students, master's students and faculty far exceeds the limited journal capacity (Wu, 2025), and the decision-making power often remains concentrated in supervisors' hands (Li, 2016), the publication space available to doctoral students has been further squeezed, resulting in their marginalization and disempowerment in the academic publishing system. Consequently, the discourse analysis shows that doctoral students have experienced emotional numbness during the prolonged life cycles of papers, and previous studies also indicate that publication pressure has become a major source of stress and anxiety (Bai et al., 2025; Horta and Li, 2023; Li, 2016).

Furthermore, under the influence of academic capitalism, knowledge production and scholarly outputs are increasingly treated as commodities (Jessop, 2018; Huang, 2021; Wu, 2025). This publication dominance encourages doctoral students to prioritize hot and publishable topics over socially and intellectually meaningful ones (Horta and Li, 2023), which threatens the sustainable development of academia and fosters scholarly opportunism. Besides, students often belittle coursework and teaching assistantships, regarding them as structural constraints or distractions that impede their research progress (Merga and Mason, 2021; Horta and Li, 2023).

### 5.4 Commodified candidates in the competitive labor market

T4 reveals how doctoral students' career planning and job-seeking practices are deeply shaped by structural transformations in higher education, academic labor market saturation, and publication-centered evaluation systems. Within this discourse, doctoral students are constructed as strategic yet precarious actors who must manage academic capital for self-packaging and self-promotion in order to survive within increasingly uncertain employment environments. Meanwhile, T4 also reflects a broader shift from traditional academic aspirations toward diversified professional paths driven by shrinking opportunities, unstable prospects, and degree inflation. Therefore, T4 simultaneously constructs doctoral identity as commodified competitive subjects in the academic labor market and as individuals continuously negotiating their future possibilities under conditions of structural uncertainty and changing employment ecologies.

T4 discursively constructs a grim reality that the supply of PhD graduates gradually exceeds available academic positions, not only in China (Jing et al., 2026; Gu et al., 2018; Chen, 2021), but also in the global academic labor market (Huisman and Naidoo, 2006; Bekova and Dzhafarova, 2019). As doctoral education expands, the academic labor market has reached a state of saturation, resulting in degree depreciation and employment difficulties (Servage, 2009; Shen et al., 2018; Bai et al., 2025). Under such conditions, the pervasive employment anxiety among doctoral students is not merely an individual psychological issue, but rather a consequence of broader institutional transformations and structural imbalance. Although many studies have shown a preference for academic positions (Huisman et al., 2002; Gemme and Gingras, 2012), doctoral students become acutely aware that staying in academia is no longer guaranteed and the proportion of doctoral graduates securing university positions is not high (Castelló et al., 2015). The increasing scarcity of academic positions, worsening job security, and high institutional requirements have prompted many doctoral students to reconsider academic careers and explore non-academic alternatives (Shen et al., 2018; Gu et al., 2018; Bao et al., 2020; Chen, 2021).

Furthermore, the analysis of T4 reinforces the findings of T3, demonstrating how publication dominance extends beyond doctoral training into doctoral students' career decision-making and employment opportunities. These findings are consistent with existing research showing that publications are increasingly institutionalized as graduation requirements and the primary academic capital in academic recruitment and promotion (Lu, 2022; Horta and Li, 2023; Jing et al., 2026). Under this regime, Horta and Li (2023) report that students with good publication profiles are optimistic about securing academic employment, while those with unsatisfactory or no publication records experience profound career anxiety. This publication-driven employment logic further consolidates academic capitalism, in which research outputs are rendered as marketable assets and doctoral students are encouraged to package themselves as competitive, publication-measured commodities.

## 6 Conclusion

This study investigated Chinese doctoral students' self-disclosure on Xiaohongshu to explore how they frame doctoral experiences and construct identities. By analyzing 9,903 posts utilizing LDA topic modeling, four major topics were identified: supervisor dominance and survival narratives, psychological distress and existential dilemmas, competence formation and academic production, and career planning and job-seeking preparation, thereby addressing RQ1. In relation to RQ2 and RQ3, recurrent discursive patterns within the examined corpus indicate that the construction of doctoral identity is a multi-layered dynamic process deeply shaped by power asymmetries within academic hierarchies, affective struggles, publication-centered evaluation systems, and uncertain employment prospects. The discourse also demonstrates doctoral students' proactive meaning-making practices, including emotional disclosure, cognitive reframing, professional identity construction, and strategic career planning, reflecting how social media platforms function as spaces for emotional catharsis, discursive resistance, and collective identity formation.

However, several limitations should be acknowledged. First, as previous research suggests that social media self-disclosure often operates as a coping strategy triggered by stressful events (Stiles, 1987; Saha et al., 2025), the engagement-ranked corpus may underrepresent those with ordinary or positive doctoral experiences while favoring emotionally intense, controversial, or algorithmically amplified posts. Reliance on only two hashtags, exclusion of visual and video content, and the lack of independently verified user status and demographic metadata further constrain its representativeness. Second, this study focused exclusively on post titles and texts, leaving discursive interactions in the comment section unexamined. Third, as a corpus-assisted discourse analysis, this study primarily explored the thematic substance of doctoral social media self-disclosure rather than verifying its actual psychological effects on the doctoral community (whether alleviating or exacerbating). Fourth, the LDA model estimation was conducted on a single fixed run rather than evaluated through repeated estimations using alternative random seeds or parameter specifications. Therefore, future research could employ repeated-run stability assessments and systematic sensitivity analyses to evaluate the robustness of the LDA analysis, triangulate by integrating cross-platform data with longitudinal experimental designs (e.g., surveys and interviews) to assess the mechanisms linking doctoral social media disclosure to their mental wellbeing based on an expanded sampling, or extend the inquiry by analyzing how doctoral narratives are validated, endorsed, questioned, and challenged by different audiences in post comments, so as to investigate how internal group identity and external public perceptions are co-constructed or contested in online doctoral communities.

## Data Availability

The original contributions presented in the study are included in the article/supplementary material, further inquiries can be directed to the corresponding author.

## Footnotes

1 LDA parameter specification. LDA topic modeling was implemented using the LatentDirichletAllocation function from scikit-learn (1.8.0) in Python. A document-term matrix was constructed using CountVectorizer, which excluded tokens occurring in fewer than 10 posts (min_df = 10) or more than 80% of posts (max_df = 0.8) and specified a vocabulary size of 2,000 top features (max_features = 2,000). Models with one to six topics were estimated using batch variational Bayes (learning_method = “batch”) with a maximum of 100 iterations (max_iter = 100) and a fixed random seed of 0 (random_state = 0) to ensure computational replicability. All other applicable parameters (e.g., doc_topic_prior and topic_word_prior) remained at their default settings.

2 According to Figure 1, perplexity decreased substantially as the number of topics increased from one to four, indicating a marked improvement in model fit, while at the elbow point four, additional topics only result in limited gains in statistical fit and potential semantic redundancy.
